# Supplementary material for: Are carbon emissions trading and green financial instruments synergistic? -Comprehensive quantitative research based on content analysis
Source: PLoS One. 2024 Mar 7;19(3):e0298601. doi: 10.1371/journal.pone.0298601 (PMC10919601; doi:10.1371/journal.pone.0298601)
Supplement: S1 Data — (ZIP) [file pone.0298601.s001.zip › Data/地级市Z_F工作报告.zip!/║╙▒▒/└╚╖╗╩╨/└┤╫╘╣1⁄2╓┌║┼í╛┬φ┐╦╩2╛▌═°í┐_2009.docx]

2008年：经济平稳较快发展社会和谐稳定

刚刚过去的一年是很不寻常的一年。面对复杂多变的国内外形势和诸多不利因素，我们在省委、省政府和市委的正确领导下，紧紧围绕“科学发展、进位争先、和谐安定、富民强市”的发展任务，迎难而上，创新举措，积极应对各种困难和挑战，较好地完成了市五届人大一次会议确定的目标任务。

——经济保持了平稳较快发展。

全市地区生产总值完成1051.5亿元，同比增长11.8%。全部财政收入完成122.1亿元，增长27.8%，其中地方一般预算收入57.9亿元，增长27.5%。主要经济指标增速保持全省前列水平。地方税收增幅居全省第一位，全部财政收入、地方一般预算收入、城镇固定资产投资、规模以上工业增加值、城镇居民人均可支配收入、社会消费品零售总额六项指标增速居全省第二位。

——经济发展方式转变取得新进展。

“项目质量提高年”深入推进。全市实施投资千万元以上项目1900项，完成投资660亿元。其中列入省重点建设项目133项、重点产业支撑项目4项，数量均居全省第一。新增计划用地指标和指标使用速度均居全省前列。

产业结构进一步优化。三次产业比例达到12.3:56.6:31.1，先进制造业、现代服务业和新型农业的支撑作用明显提升，传统优势产业优化升级、新兴产业快速成长的局面正在形成。电子信息产业基地形成框架，集群效应开始显现，销售收入同比增长94.7%。装备制造业快速发展，首钢装备制造业基地、瑞斯通数控机床、富邦德石油钻井机械等重点项目加快建设，正在成为产业发展的新亮点。金融保险、会展旅游、物流等现代服务业迅速壮大。新型农业发展步伐加快，特色农业、观光休闲农业规模继续壮大，林业发展保持全省领先，畜牧业产值占农业总产值比重达到38%，农业综合开发投入1.4亿元。新增省级重点龙头企业11家，农业产业化经营率达到62%。

园区承载力得到增强。切实加强园区建设，促进项目集中、资源集约、企业集聚。新增4个省级园区，建设了一批乡镇产业功能区和“区中园”，启动了对接天津滨海新区的霸州津港工业园。固安工业区获得中国最具投资价值十大环保园区称号。10个重点园区完成财政收入、实际利用外资分别占全市的50.6%和90.5%。

节能减排力度加大。全市新建污水处理厂5座。单位生产总值能耗下降4.6%，二氧化硫、化学需氧量排放量分别下降9.5%和6.7%，超额完成省下达的目标任务。市区空气质量持续改善，二级以上天数达到339天。我市连续9年成为省环保目标考核优秀市。

——城乡建设和管理水平全面提升。

城镇面貌三年大变样工作扎实开展。市区完成拆迁207.2万平方米，拆墙透绿1.67万延长米，拆迁量相当于过去10年的总和。投资3700万元，编制了空间发展战略研究、京沪铁路两侧控制性规划、生态居住小区细则等专项规划，金光道景观规划方案获全省最佳方案。完成市区7条道路建设和改造，实施了金光道、环城水系、郊区快速路景观改造工程，南出口、西出口、西外环南延和永兴路铁路桥工程竣工。对2个城中村和2个棚户区实施了拆迁改造，完成了26个旧住宅小区拆迁改造任务。市区新增绿化面积200万平方米，绿化覆盖率达到46%，人均公共绿地面积12.1平方米。

新农村建设成效显著。村庄整理和新民居建设试点顺利推进，启动建设32个村庄整理试点，整理出建设用地3786亩，规划新民居建设村街677个，7个试点村建成新型农民住宅16.9万平方米。启动第四批150个文明生态村街创建，建设示范性村民中心100个。永清县、霸州市被命名为全省新农村建设工作先进单位。

城乡基础设施建设实现突破。廊涿高速竣工通车，廊沧、大广高速开工建设，京台和密涿支线高速前期工作顺利推进。廊涿高速九州连接线、京沪高速连接线大城段竣工。新建改造农村公路440.8公里，改造县乡道路危桥13座。建成两个220千伏和四个110千伏输变电工程。南水北调配套工程、文安防洪安全区、IGCC热电联产等项目取得阶段性成果，对接北京轨道交通工作取得初步进展。

——改革开放扎实推进。

国企改革继续深化，市政二公司、市运输公司完成改制，通洲筑路、新宇纺织等5家企业正在进行改制后扫尾工作。全民创业成效显著，全市民营经济完成增加值601亿元、上缴税金75亿元，分别增长8.8%和32.3%；建成创业辅导基地17个，入驻企业341家。市商业银行股权改制取得实质进展，全市农村信用社兑付中央银行专项票据16.5亿元，44家小额贷款公司累放贷款44亿元，18家担保公司累计担保金额32.4亿元。对外开放不断加强，全市实际利用外资4.2亿美元，增长21.1%；“5·18”投洽会、“9·26”农交会成果丰硕，“12·16”投资环境推介会在北京成功举办。

——改善民生工作进一步加强。

城乡居民收入不断提高，全市城镇单位在岗职工平均工资增幅在全省领先，城镇居民人均可支配收入16302元，农民人均纯收入6155元，分别增长16.7%和9.9%。新增就业岗位5.1万个，城镇登记失业率2%。1054名残疾人实现就业，326户贫困残疾人住房条件得到改善，城镇"零就业家庭"保持动态归零，在全省率先建立孤儿救助长效机制，适龄孤儿全部免费就读。五保户集中供养率达81.2%，继续保持全国领先。社会保险覆盖面进一步扩大，廉租住房保障从最低收入家庭扩大到低收入家庭。投资1.1亿元改造625所中小学取暖设施，改扩建校舍5.5万平方米。农村义务教育阶段学杂费、书本费全部免除。全市302个村、28.8万人的饮水安全问题得到解决。

——社会事业取得新成绩。

我市成为全省首家国家可持续发展实验区，新增1家国家级生产力促进中心，新建7家市级企业技术研发中心，高新技术产业增加值突破百亿大关，同比增长31.5%。河北省(廊坊)电子信息职教集团组建完毕，廊坊职业技术学院通过国家验收，各县(市)都建成了省级示范性普通高中。省运会筹备工作进展顺利。文化信息资源共享工程乡镇覆盖率居全省第一，文安县被评为全国基层文化网络建设先进县。县乡村医疗卫生服务体系标准化、规范化建设在全省率先启动。低生育水平保持稳定，人口自然增长率控制在5.02%。。安全生产主要控制指标连续5年保持全省领先水平。

——政府职能加快转变。

加大清理、削减行政审批事项和收费项目力度，开通行政审批电子监察系统，50个单位、398项行政审批事项纳入电子监察范围。开通“1890”投诉电话，群众诉求受理机制进一步完善。制定出台行政首长问责暂行办法，切实维护政令畅通。政府法制工作加强，依法行政水平不断提高。自觉接受人大和政协的监督，办理市人大代表建议129件、市政协提案387件，按时办复率为100%。主动听取各方面意见，接受社会各界监督。

2009年主要目标：地区生产总值增长10%以上

政府工作报告提出，今年政府工作的总体要求是：全面贯彻党的十七大和十七届三中全会精神，以邓小平理论和“三个代表”重要思想为指导，深入贯彻落实科学发展观，把保持经济平稳较快发展作为首要任务，把转变发展方式和结构调整作为主攻方向，把深化改革开放作为强大动力，把改善民生作为出发点和落脚点，聚焦“京津冀电子信息走廊、环渤海休闲商务中心”发展定位，推进“科学发展、进位争先、和谐安定、富民强市”的发展任务，迈出实现“两个率先”奋斗目标的坚实步伐。

今年我市经济社会发展的主要目标是：地区生产总值增长10%以上；全部财政收入增长15%以上，其中地方一般预算收入增长15%以上；全社会固定资产投资增长20%，其中城镇固定资产投资增长22%；规模以上工业增加值增长15%以上；城镇居民人均可支配收入增长10%以上，农民人均纯收入增长8%以上；单位生产总值能耗下降5.6%，二氧化硫排放下降3%，化学需氧量排放下降5.5%。

实现今年经济社会发展的主要目标，重点突出“两抓一保”：抓好现代产业体系培育。以项目建设为核心，优环境，增投入，促进产业结构升级，着力构建以先进制造业、现代服务业、新型农业为主导，以传统优势产业为基础的金字塔型产业体系。抓好现代城镇体系建设。深入推进三年大变样工作，加快城乡一体化步伐，努力形成中心城市率先发展、县城和重点镇协调推进的发展格局。确保民生改善、公共安全、社会稳定三大基础。

增投入，上项目，千方百计保增长

大力做好投资和项目工作。按照以项目增投资、以投资促发展的思路，扎实推进项目建设。围绕现代农业、高新技术产业、现代服务业、传统产业升级、基础设施建设等领域，实施建设超亿元项目300个以上，确保城镇固定资产投资达到1000亿元以上。

推进一批投资规模大、产业链长、带动面广的龙头项目。全力做好富士康、华为、中兴、新奥光伏、首钢装备制造业基地等在建项目服务工作，促其加快建设进度，尽早投产达效。对大厂金隅、宇信和固安峻凌等项目，加紧落实开工条件，争取尽早开工建设。积极争列和实施省重点项目，扎实做好前期工作，力争省重点项目数量继续保持全省领先。强化项目调度，与各县(市、区)签定责任状，对已供地不按时开工、已开工达不到进度要求的实行问责制。

谋划实施一批具有战略意义的基础设施项目。积极争取更多项目列入国家投资计划。实施一批重大交通项目。廊沧高速完成主线征地拆迁，路基、桥涵及东淀特大桥桩基工程完成70%以上，京台、密涿支线高速开工建设，廊涿高速固安、永清连接线、唐通线北京至香河段等工程竣工通车。加快南水北调相关工作进展，廊坊干渠力争年内开工建设。建设一批电力工程项目。力争IGCC热电联产项目开工，完成市区热电联产规划，谋划市区第二电源点；建成一个220千伏、三个110千伏输变电工程。

全力引导和帮扶企业。最大限度地减免各类行政事业性收费，减轻企业负担。制定特殊时期中小企业扶持政策，放宽出资期限、停业时间、年检材料等方面的要求，灵活采用提示、警告、告诫、责令改正等软性执法行为。引导关联企业、上下游企业联合协作，形成资源、资金、市场、技术的整体优势。进一步理顺经营性国有资产管理体制，有效防范国有企业经营风险。定期举办银企对接洽谈活动，努力为企业创造宽松的融资环境。以“团结互助、诚实守信、尽职敬业、奉献社会”为核心内容，积极打造廊坊商业文化。

进一步增强园区承载力。原有省级园区抓好规划提升，加强基础设施改造升级，大力引进研发机构、大型企业地区性总部，积极培育高端服务业；新批省级园区坚持高标准规划，重点加强道路、商住、环保、金融等服务设施建设。创新园区运行机制，廊坊开发区、燕郊开发区探索新模式，管理与开发分离，强化管委会公共管理职能，实施以公司为主体的市场化开发。利用北京新机场建设的机遇，全面启动廊坊临空经济区规划工作，聘请权威咨询机构，系统深入研究。上半年完成临空经济区规划编制，年底前完成向省政府申报工作。

全力推进全民创业。全市民营经济增加值增长12%，上缴税金增长15%，新建担保机构3家、创业辅导基地4个，完成各类创业培训18万人次。研究出台促进企业二次创业、联合创业、互助创业的政策措施。

切实优化软环境。大幅度清理和削减行政审批事项和收费项目，努力实现全省最少的目标。全面推行服务承诺制、首问首办责任制、限时办结制，为企业提供高效快捷的服务。规范行政执法行为，规范行政处罚自由裁量权。实施最严厉的问责制，对各种损害发展环境的行为，严厉追究行为责任人和主管领导责任。

推进产业结构优化升级，提高可持续发展能力

加快发展先进制造业。壮大电子信息产业，做强生物医药产业，发展装备制造业，培育装备制造产业集群。

积极发展现代服务业。重点发展现代物流、会展旅游、文化创意、健康服务等休闲商务产业。

大力发展新型农业。重点发展畜牧养殖、绿色蔬菜、林果花木和休闲观光农业。大力发展双节双高农业。农业产业化重点抓好“四个十”：做大10个市级标志性龙头企业；做强10个市级种植业基地和10个养殖业基地；建设粮油、林板、肉制品等10个优势产业集群，销售收入达到300亿元。农业产业化经营率达到63%。

推进自主创新和产业升级。以科技谷、燕郊东湖孵化器、清华研究院孵化器等为重点，引进科技机构和人才，提高创新能力。加快建设农村信息化工程中心、燃料电池、煤矿安全、内燃机等省级重点实验室，推动新奥能源实验室等企业研发中心建设，增强自主研发能力。出台促进传统产业升级的政策措施，鼓励企业引进新技术、新工艺、新装备，提高产品技术含量和附加值，培育知名品牌。

打好节能减排攻坚战。对各县(市、区)实施目标管理。深入实施“1225”减排工程。加大污水和垃圾处理力度，5月1日前，所有县(市)的污水处理厂投入运行，力争建成万庄和市区第二个污水处理厂，在各省级园区和50%的省级重点镇建设污水处理厂，实现县县有垃圾处理场目标。开展市区、县城及重点镇的空气治理，实施龙河、潮白河、中亭河等六大流域综合治理。全面推进生态市创建工作，三河、霸州创建省级环保模范城市，力争建成1-2个国家级环境优美城镇。

以三年大变样为抓手，促进城乡建设协调发展

强力推动市区三年大变样。立足政府主导，拆建并重，实施大拆迁、大融资、大招商、大建设。政府主导性投资100亿元，拉动社会投资400亿元，完成拆迁200万平方米，打好拆迁整治、老城和城中村改造、新城区整体开发三大战役，实施12项城市精品工程。

推进重点工程建设。加快万庄生态城和科技谷建设。启动光明西道片区改造、龙河商务中心、开发区商务中心、明珠商业中心建设。建设贯穿市区的迎宾大道。谋划启动“大三点”组团快速交通通道。加快市区水系、自然公园二期、廊坊植物园、生活垃圾发电等项目进程。全面启动城中村改造，拆迁150万平方米。加快高架桥西侧和李桑园西口棚户区拆迁改造。实施旧小区改造20万平方米。

切实做好重点工作。高标准做好交通路网、供水排水、供热供气、绿化亮化等专项规划，编制市区重要路段、街区、节点的控制性详规。

提高城镇建设水平。坚持市县联动，推动三年大变样向城镇延伸，确保城镇面貌明显改观。实施“穿衣戴帽”工程，加强县城和重点镇主街主路、节点、出入口的综合改造提升，每个县城建成2条样板街道。完善城镇功能，重点加强交通、电力、供热、供水排水、污水处理等基础设施建设。抓好县城和重点镇总体规划修编，努力实现县域控制性详规全覆盖。

进一步改善农村面貌。积极稳妥地推进新民居建设，每个乡镇建成20个示范户，每个县(市、区)新建3个样板村，全市完成30个。扎实推进村庄整理，年内重点推进15个村。加快农村改革步伐，以县级服务组织为平台、乡级服务站点为依托，建立健全土地承包经营权流转市场。大力实施“城乡绿化再创辉煌大会战”，新增造林30万亩。加快农村基础设施建设，新建、改造公路380公里，新增节水灌溉面积10万亩，新打机井1000眼。

以全面对接京津为突破口，再创发展新优势

构建合作平台。加强与京津的沟通联系，形成稳定的对话渠道，建立政府机构合作平台。以13个省级园区和万庄生态城、科技谷为载体，积极与京津政府机构、园区、大企业大集团沟通联系，积极争取产业链接。探索园区共建，支持各县(市、区)划出部分土地，与京津合作开发，统筹摆放项目。

实施城市整体营销。以北京为重点，以驻京国际大公司、国家大集团、北京大企业为主要对象，细分各类企业需求，量身定做营销计划，开展大规模、高密度的宣传攻势，打出京廊同城概念，扩大北京新机场及京廊轻轨建设的影响，整体提升廊坊城市品牌。

加强规划对接。与京津规划部门进行全面对接，尽快编制京津廊发展合作的规划衔接图。

加快交通对接。把京廊轻轨建设作为重中之重，全力做好L2线延伸至市区、八通线延伸至燕郊项目的前期工作，尽快达到申报条件，力求早日立项、早日开工。启动实施大厂对接通州的新潮白河大桥建设，提早谋划北京新机场连接线工程。

推进产业对接。建设四大基地：即农副产品生产基地、项目转移基地、产学研基地和休闲商务基地。努力建成京津大公司、大机构和成功人士休闲办公的首选之地。

大力推进以改善民生为重点的社会建设

政府工作报告明确，2009年我市将大力推进以改善民生为重点的社会建设，着力保障和改善民生。重点办好六方面18件实事：

把解决就业问题作为民生之基，放在首位。⑴帮助返乡农民工、“零就业”家庭、城市拆迁失业人员、大学毕业生和就业困难人员就业。

加强城乡医疗服务体系建设。⑵新型农村合作医疗实行提补、扩面，筹资标准由每人90元提高到100元，农民参合率达到85%以上。⑶全面推开城镇居民医疗保险试点，进一步扩大保险面，参保率达到80%；城镇低保对象全部纳入城镇居民医疗保险体系，个人免交保险金。⑷资助五保户、低保户和生活困难的残疾人参加新型农村合作医疗，参合率达到100%。落实好低保、五保、孤儿等困难群体定点就医、费用减免政策。⑸开展白内障万人复明工程，免费为1132名贫困白内障病人实施手术。⑹完善农村卫生服务体系，搞好“三基两化”建设，在全省率先完成乡镇卫生院、村卫生室标准化、规范化建设，让广大农民方便看病、看得起病、看得好病。⑺建立120医疗急救调度指挥中心，实现城区覆盖。

增加保障性住房供给。⑻建设经济适用房15万平方米，回购、配建廉租房1000套，解决3200户低收入家庭的住房问题。⑼开展农村低保特困户、灾贫户危房帮建工作，年内帮建1000户，争取三年全部完成。⑽充分发挥住房公积金制度的保障作用，新增归集7亿元，保障人群拓展到21万人，覆盖面达到80%以上；发放贷款5亿元，个人贷款率达到45%以上。

改善办学条件。⑾建设34所农村标准化初中、小学，改善农村教学条件。⑿完成廊坊市第十四小学、第一实验小学和管道局中学的改扩建工程。

完善社保体系。⒀加大统筹力度，力争实现基本医疗保险市级统筹，企业退休人员基本养老保险金再提高10%以上。⒁建立廊坊市社会保障服务中心，实现养老、医疗、失业、工伤、生育“五险合一”，让群众办事更方便。

提高城乡居民生活质量。⒂完善农畜产品检测中心，新建果品质量监督检测中心，保障消费者的食品安全。⒃建设100家“药品质量管理示范店”，让群众吃上放心药。⒄投资1亿元，新建10座农村水厂，解决20万人的饮水安全问题。⒅改造市区8条小街巷和7处交叉路口，让市民出行更便捷。

全力维护公共安全和社会稳定

强化食品药品安全。深入开展“食品药品安全质量提高年”活动，实施放心食品“一十百千工程”，创建1个省级重点食品检验中心、10个食品规范经营示范市场、100个城镇示范店、1000个农村示范店。开展食品药品安全隐患治理集中行动。理顺食品药品监管体制，积极争取组建广阳区、安次区食品药品监督管理机构。

加强安全生产。健全安全生产投入机制、责任追究机制，促使企业增加安全生产投入，落实生产经营单位安全主体责任。建立重大安全隐患挂牌督办制度，对排查出的各类重大安全隐患，由市、县两级政府挂牌督办，确保按期整改到位。建立市级重特大事故应急救援指挥中心和重大危险源监控中心，完善重点企业的应急救援预案。

切实维护社会稳定。坚持“三位一体”大调解机制，妥善处置土地征用、城镇拆迁等涉及群众切身利益的实际问题。高度重视信访工作，完善领导接访制度。增强突发公共事件快速处置能力，完善各类预案。大力开展平安社区、平安企业等创建活动，强化社会治安综合治理，加强流动人口和出租房屋管理，依法严厉打击违法犯罪活动。

适应新形势，努力提高领导科学发展的能力

第一，提高勤俭持政的能力。大力削减大型会议，严格控制公务外出考察团组，压缩公务接待费，冻结公务用车购置，一般性行政费用支出零增长，大力开展节约型机关建设。

第二，提高攻坚克难的能力。通过创新举措解决长期积累的问题和深层次矛盾，以重点、难点问题的突破带动全局、活跃全局。

第三，提高抓落实的能力。在全市公务员队伍中开展“作风建设年”活动，全面实施行政问责制，有效保障工作落实。

第四，提高依法行政的能力。严格按照法定权限和程序行使权力，履行职责。自觉接受人大法律监督和政协民主监督，认真倾听社会各界的意见。加强对权力运行的监督，保障行政权力公开透明运行，从源头上预防和治理腐败。严肃法纪，坚决纠正损害人民群众利益的不正之风，严厉查处违法违纪案件。
